# QYAG5 Q-switched Nd:YAG Laser Treatment of Nevus of Ota: An Indian Study of 50 Patients

**DOI:** 10.4103/0974-2077.44164

**Published:** 2008

**Authors:** Sanjeev Aurangabadkar

**Affiliations:** *Consultant Dermatologist and Laser Surgeon, Skin and Laser Clinic, Hyderabad, Andhra Pradesh, India*

**Keywords:** Nevus of Ota, Q-switched Nd:YAG laser, multiple treatment

## Abstract

**Background:**

Nevus of Ota is very common in Asian patients. The condition is more common in females, with a male-female ratio of 1:4.8. Most patients seek treatment early in life due to the psychological trauma and cosmetic disfigurement. The Q-switched lasers have changed the way we approach the condition and have become the mainstay of therapy.

**Aims and Objectives:**

To evaluate long-term safety and efficacy of pigmented lesion laser Palomar QYAG5 Q-switched Nd:YAG in 50 Indian patients.

**Materials and Methods:**

Fifty patients of nevus of Ota underwent multiple treatments (average 6 sessions) carried out over a period of 1year with a Q-switched Nd:YAG laser (QYAG5, Palomar, USA). Of the 50 patients, 2 were males; and the rest, females. Five patients had a bilateral involvement. Skin types treated included phototypes 4 and 5. The response after subsequent treatments was documented through serial photographs that were taken before and after the completion of treatments. Patients were followed up for a period of 1 year after the last session. Response to treatment was graded based on physician’s global assessment.

**Results:**

Excellent improvement was noted in a majority of the patients at the end of the treatments. Greater-than-60% improvement was seen in 66% of the patients. The remaining patients had moderate clearing of pigmentation (30%-60% improvement). No significant adverse effects were seen immediately after the treatments and on long-term follow-up. Transient post-inflammatory hyperpigmentation was observed in 5 (10%) patients, which cleared with use of sunscreens and bleaching agents within 2 months. No textural change or scarring was seen. Hypopigmentation (guttate type) was observed in 1 (2%) patient, which resolved within 3 months. No recurrence was observed after 1 year of follow-up.

**Conclusion:**

This study validates the superior efficacy of Q-switched Nd:YAG laser when compared to conventional methods for treatment of nevus of Ota.

Nevus of Ota (oculodermal melanocytosis, nevus fuscoceruleus ophthalmomaxillaris) is a dermal melanocytic hamartoma that presents as bluish/slate-gray hyperpigmentation along the first or second branch of the trigeminal nerve. In 1916, Pusey described the case of a Chinese student with both scleral and facial pigmentation.[1] It was not until 1939 that the condition was defined as an entity by Ota, from the University of Tokyo, who described bluish-gray irregular hyperpigmentation along the first and second divisions of the trigeminal nerve with frequent mucosal involvement. Since then, this melanocytic nevus has been widely known as nevus of Ota.

Nevus of Ota is the commonest in Asian patients and affects between 0.014% and 0.034% of the Asian population. The age of onset is bimodal, with larger peak at birth or soon after and a smaller peak at adolescence. Nearly all lesions appear by 30 years of age.[2] It is usually unilateral on areas supplied by ophthalmic and maxillary divisions of trigeminal nerve, predominantly V1 and V2. This usually corresponds to forehead, temple, nose, eyelid, ear, and scalp.[2] It may involve the sclera, with hyperpigmentation of the cornea, iris, retina, hard palate.[2] The pigmentation varies and can be dark brown to blue to black-blue.[3,4] The condition is more common in females, with a male-female ratio of 1:4.8.

Most patients seek treatment early in life due to cosmetic disfigurement and the ensuing psychological trauma. Treatment options were limited prior to the advent of laser therapy. The Q-switched lasers have changed the way we approach the condition and have become the mainstay of therapy. We aimed to study the safety and efficacy of Nd:YAG laser in Indian population.

**Aim of the study**

The aim of the study was to document the safety and efficacy of Nd:YAG laser in Indian patients.

## MATERIALS AND METHODS

Fifty patients of nevus of Ota underwent multiple treatments carried out over a period of 1 year with a Q-switched Nd:YAG laser (QYAG5, Palomar, USA). Table 1 shows the demographic profile of the patients. Of the 50 patients, 2 were males; and the rest, females. Most patients were in the age group 21-25 years, with mean age being 23 years. Five patients had a bilateral involvement. The youngest patient treated was 18 years old, while the oldest was 39 years old. Skin types of the patients treated included phototypes 4 and 5.

**Table 1:** Demographic profile of patients

Males	Females
2	48

**Unilateral**	**Bilateral**

45	5

**Age Group**	**Number of patients**

16-20 years	6
21-25 years	32
26-30 years	8
31-35 years	2
36-40 years	2

Patients were advised to use a sunscreen at least 15 days prior to the onset of laser therapy and throughout the duration of treatment. Bleaching creams containing hydroquinone or kojic acid were used if the patients had tanned skin. Topical anesthesia with eutectic mixture of lignocaine and prilocaine was used 2 hours prior to treatment under occlusion if the lesion being treated was a large one. Otherwise, no anesthesia was necessary. All personnel in the room wore appropriate eye protection during laser treatment. During treatment, the patients wore eye shields provided by the manufacturer. The area within the orbital rim was not treated. Therapy was initiated with 1064-nm Q-switched Nd:YAG laser at fluences of 2 to 2.5 joules/cm^2^, the end point being immediate whitening of the lesion on laser irradiation. Since this dosage did not yield any significant response, the fluence was subsequently increased to 3-3.45 joules/cm^2^. A maximum fluence of 3.45 J/cm^2^ at 4-mm spot size, which was permissible by the laser, was used for the rest of the sessions in all cases. In those cases where residual pigmentation was observed after 6 sessions, the fluence was increased to 7-8.5 J/cm^2^ by reducing the spot size to 2 mm. The wavelength used was 1064 nm with an average fluence of 3.45 J/cm^2^ at 4-mm spot size, 7 J/cm^2^ at 2-mm spot size; and 10 Hz repetition rate. The pulse duration was 3 ns. Of the patients treated, 43 patients received a maximum fluence of 3.45 J/cm^2^ at 4-mm spot size; and in the remaining 7 patients, the fluence was increased to 7-8.5 J/cm^2^ at 2-mm spot size after the sixth session.

Treatment was performed with the hand piece held perpendicular to the skin surface with minimal overlap (about 10%). The entire lesion was covered with a single pass. On an average the interval between laser treatments was 2 months. All patients completed a minimum of 6 treatments and were followed up for 1 year postoperatively.

The response after subsequent treatments was documented through serial photographs that were taken before and after the completion of treatments. The patients were followed up for a period of 1 year after the last session. Response to treatment was graded as (based on physician’s global assessment) –

Excellent: Greater than 60% improvement

Moderate: Between 30% and 60% improvement

Poor: Less than 30% improvement (clearing)

## RESULTS

**Figure 1:**
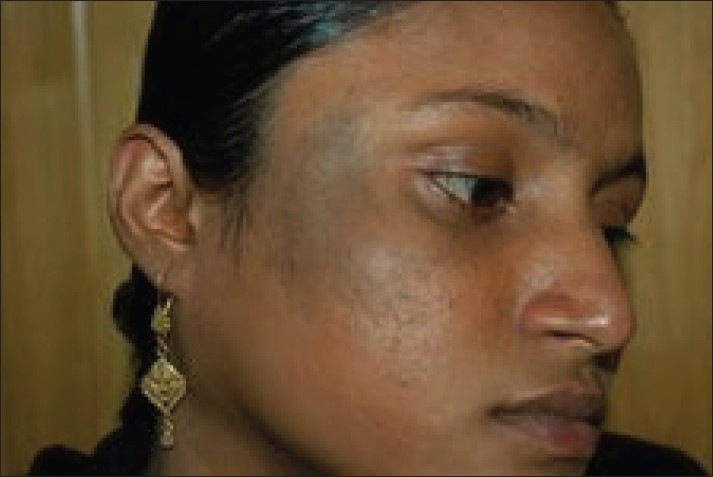
Patient with nevus of Ota on right maxillary distribution before treatment

**Figure 2:**
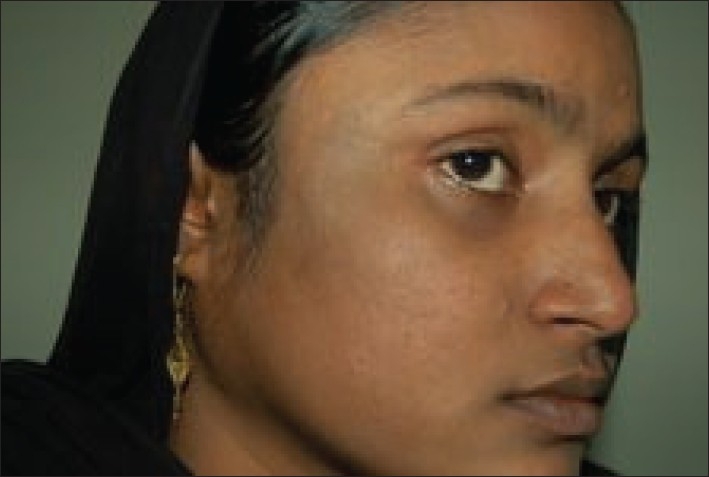
Patient with nevus of Ota on right maxillary distribution after 6 treatment sessions

**Figure 3:**
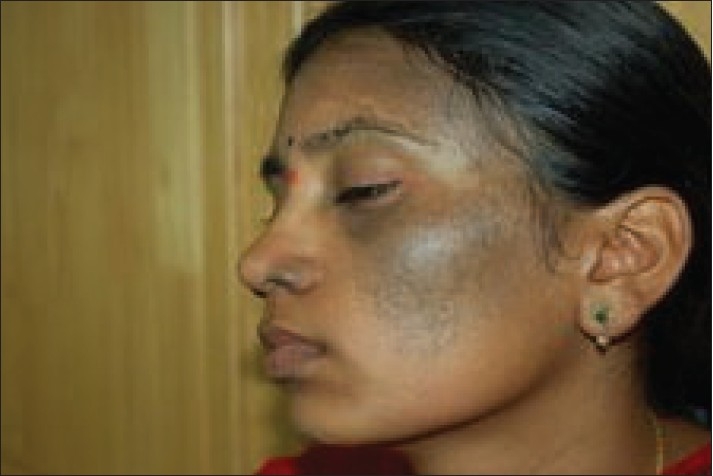
Patient with bilateral nevus of Ota (left side) on temporal and maxillary distribution before treatment

**Figure 4:**
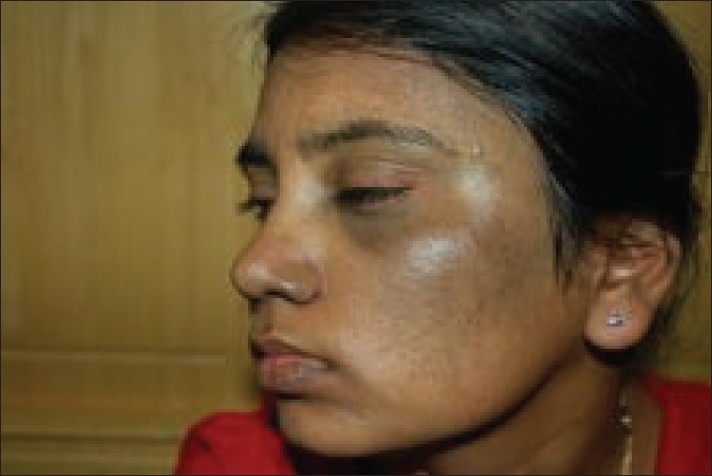
Patient with bilateral nevus of Ota after 6 sessions

**Figure 5:**
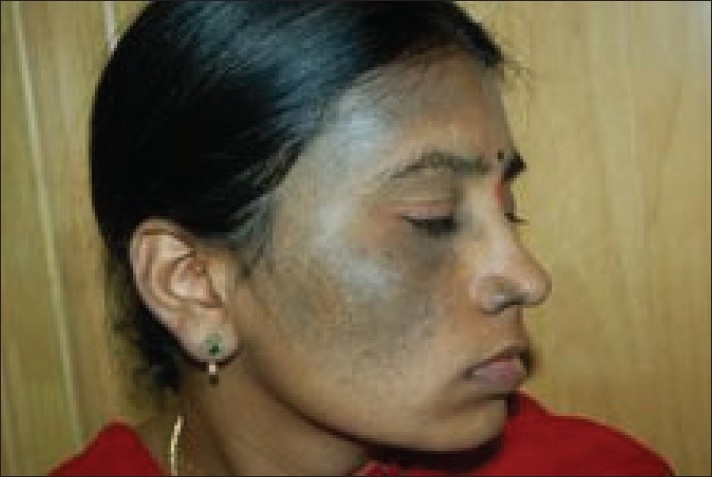
Patient with bilateral nevus of Ota (right side) on temporal and maxillary distribution before treatment

**Figure 6:**
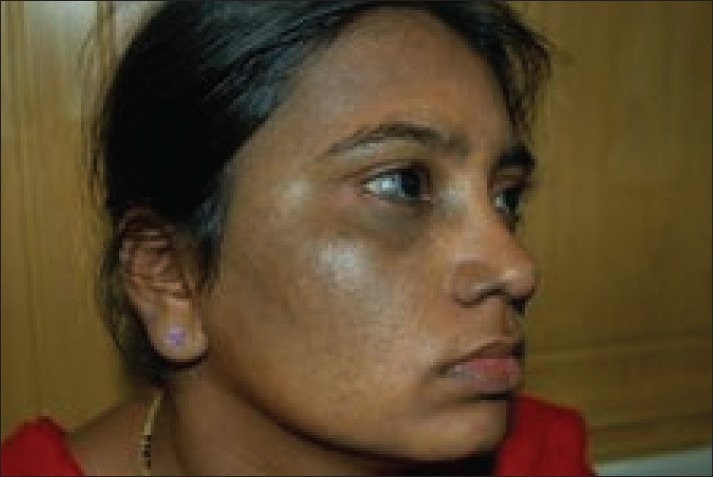
Patient with bilateral nevus of Ota after 6 sessions

Most patients showed some improvement, and none showed poor response. Excellent improvement was noted in a majority of the patients at the end of the treatments. Greater-than-60% improvement was seen in 66% of the patients. The remaining patients had moderate clearing of pigment (30%-60% improvement). Use of higher fluences gave better results, with most patients responding to the highest allowed energy of 3.45 J/cm^2^ at 4-mm spot size. Use of a spot size of 2 mm was also associated with good clearing of pigment but with higher complication rate. The patients were followed up for 1 year after the last treatment session. On continued follow-up, it was observed that the pigment continued to lighten. No significant adverse effects were seen immediately after the treatments and on long-term follow-up. Transient post-inflammatory hyperpigmentation was observed in 5 (10%) patients, which cleared on use of sunscreens and bleaching agents within 2 months. No textural change or scarring was seen. Hypopigmentation (guttate type) was observed in 1 (2%) patient, which resolved within 3 months. No recurrence was observed during the 1-year follow-up [Figures 1-6].

## DISCUSSION

Prior to the advent of lasers, the treatment options for this condition included cryotherapy,[5] dermabrasion,[6] surgical excision,[7] and cosmetic camouflage. The Q-switched lasers have been used successfully to treat nevus of Ota.[8]

The Q-switched lasers produce ultra-short bursts of laser light that specifically targets the melanosomes in the dermal melanocytes.[9,10] The pulse duration of these lasers is typically in nanoseconds, and it closely matches the thermal relaxation time of the target (melanosomes).[11] The Q-switched laser treatment is based on Anderson and Parish’s theory of selective photothermolysis.[12] This states that the laser light must be of a wavelength that is well absorbed by the target chromophore and not the surrounding structures[13,14]; the pulse duration should be equal to or less than the thermal relaxation time of the target so that the heat is confined to the target, thus preventing collateral thermal damage; and finally, sufficient fluences should be used to produce the desired effect. The laser also produces an additional photoacoustic effect.[15,16] High energy is delivered in a very short time, which leads to rapid thermal expansion of the target. This produces shock waves, which leads to explosion of the target.

Of all the Q-switched lasers available today, Ruby 694 nm, Alexandrite 755 nm, and Nd:YAG 1064 nm, the longer wavelength Q-switched Nd:YAG laser at a wavelength of 1064 nm is best suited for the treatment of darker skin types as it minimizes the risk of epidermal injury and pigmentary alteration.[16,17] This wavelength is weakly absorbed in epidermal melanin and has deeper penetration into the dermis and is ideal for the treatment in skin types 3 to 6. The newer lasers have a larger spot size, which also allows deep penetration, thereby minimizing tissue splatter and preventing textural changes.[18] The treatment of skin types 4, 5, and 6 is a challenge due to the post-op risk of hyperpigmentation and hypopigmentation. This has been minimized with the shift from shorter-wavelength lasers to longer-wavelength ones, use of large spot sizes with lower fluences, and use of sunscreens and bleaching agents in the preoperative and postoperative phases. On an average, 4 to 8 sessions are required to clear most lesions, with an interval of 2 to 6 months between sessions.[19] Lesions will continue to clear during this time, probably due to melanophages clearing the melanin from previously targeted melanocytes. The condition may also recur in patients with complete clearing after laser treatment.[20] The risk of such recurrence is estimated to be between 0.6% and 1.2%. This is particularly important for children with nevus of Ota, as early treatment has been the standard practice.[19] Recurrence is probably due to residual melanocytes that did not originally contain sufficient melanin for eradication.

Goldberg and Nychay[21] and Geronemus[22] were among the first to report the use of QS ruby lasers to treat nevus of Ota. Geronemus treated 15 patients one to seven times with the ruby laser. Four patients had 100% clearing, and the remaining 11 patients had more than 50% clearance. The clinical efficacy of the QS ruby laser (QS ruby) was later confirmed in a study of 114 nevus of Ota patients who had been treated with a QS ruby.[23] The study demonstrated that a good-to-excellent degree of lightening was achieved after 3 or more treatment sessions. The side effects were few, with transient hyperpigmentation after the first treatment being the most commonly encountered adverse effect. Hyperpigmentation in 8 patients, cleared in 2 months.

Seven patients were treated by Alster *et al.;* 2 treatments resulted in an average of 50% clearance, and 5 of the 7 patients had 100% clearance after 5 treatments.[24] No recurrences occurred after 1 year. No pigmentation changes or scarring occurred. QS Alexandrite and QS 1064 nm neodymium:yttrium-aluminum garnet (QS 1064 Nd:YAG) was also used successfully for the removal of nevus of Ota.[25]

The Palomar QYAG5, a new-generation Q-switched Nd:YAG laser, is different from the older Q-switched Nd:YAG lasers in that the laser has a very short pulse duration of 3 ns that helps in delivering higher peak power. Also, the laser has a direct beam delivery hand piece that allows a more uniform top-hat beam profile, as there is no articulated arm or fiber-optic cable to deliver the laser light. The laser also has a large spot size, which helps in deeper penetration of the laser beam, thus allowing sufficient energy to be delivered to the melanocytes in the dermis. Because of the beam characteristics, the laser produces minimal tissue splatter, thereby reducing the chances of textural changes of the skin. Also, a ‘top-hat’ beam profile produces minimal ‘hot spots’ in the tissue due to a direct beam delivery hand piece, thereby minimizing adverse effects such as burns, tissue splatter, and purpura. Finally, the 1064-nm wavelength is ideal in treating darker skin tones due to its weak absorption in epidermal melanin, thus reducing complications and scarring.

In our study, all patients responded well to laser treatment, with majority showing excellent clearing. Moderate improvement was seen in 33% of the patients. This is in conformity with previous reports mentioned earlier. The use of a large spot size, direct beam delivery of laser light, very short pulse duration, and longer wavelength has multiple benefits. One, the large spot size and longer wavelength allow deep penetration of laser, thereby eliciting adequate tissue response at the desired depth in the dermis. Two, a ‘top-hat’ beam profile produces minimal ‘hot spots’ in the tissue due to a direct beam delivery hand piece, thereby minimizing adverse effects such as burns, tissue splatter, and purpura. Three, the 1064-nm wavelength is ideal in treating darker skin tones due to its weak absorption in epidermal melanin, thus reducing complications and scarring.

Studies comparing the use of QS Alexandrite and QS 1064 Nd:YAG lasers found that most patients showed a better tolerance for the former rather than the latter as a treatment modality.[26] However, the QS 1064 Nd:YAG laser was shown to be more effective than the QS Alexandrite in the lightening of nevus of Ota after 3 or more laser treatment sessions.[27]

Hypopigmentation is a commonly reported complication, especially among those who are treated with the QS ruby.[28,29] The alternate use of QS Alexandrite and QS 1064 Nd:YAG in different treatment sessions was also associated with a higher risk of complications.[28] Complications included hypopigmentation (15%), hyperpigmentation (3%); and textural changes (3%), scarring (2%). The Nd:YAG laser required more sessions to achieve a 50% clearing than did the ruby laser. A retrospective study by Kono and Mikashima[30] looked at the long-term complications of Q-switched ruby laser treatment in 101 patients of nevus of Ota. One patient had a recurrence, 17% had hyperpigmentation, and 6% had hypopigmentation. In our study, only 10% of the patients developed post-inflammatory hyperpigmentation. This was seen mostly in those patients in whom higher fluences (7-8.5 J/cm^2^) and smaller spot size (2 mm) were used. Hypopigmentation was observed in only 1 patient, probably due to the use of very high fluences to clear residual pigmentation. Hypopigmentation, reported earlier after treatment was seen mainly in some patients in whom shorter wavelengths were used (ruby, 694 nm; and Alexandrite, 755 nm).[28–30]

Recurrence has been noted, even after complete clearing after laser treatment.[20] No recurrence was found in our patients, possibly because the follow-up period was short. Hence our patients need longer follow-up period to determine possible recurrence.

## CONCLUSION

QYAG5 laser is an effective and safe treatment modality for nevus of Ota in Indian patients of phototypes 4 and 5. Side effects are few and mild.
